# Associations of sleep quality, quantity and nutrition in oldest-old men The Helsinki Businessmen Study (HBS)

**DOI:** 10.1007/s41999-020-00421-z

**Published:** 2020-11-01

**Authors:** Satu K. Jyväkorpi, Annele Urtamo, Mika Kivimäki, Timo E. Strandberg

**Affiliations:** 1 Helsinki University Hospital, Clinicum, University of Helsinki, Helsinki, Finland; 2 Center for Life Course Health Research, University of Oulu, Oulu, Finland

**Keywords:** Sleep quality, Sleep quantity, Nutritional status, Vegetable intake, Fish intake, Oldest-old men

## Abstract

**Introduction:**

Sleep quality and quantity often decline as people age, which may negatively impact health. We examined how nutrition is associated with self-reported sleep quality and quantity in oldest-old community-dwelling men.

**Methods:**

In this cross-sectional analysis of the Helsinki Businessmen Study (HBS), a random sample of 130 surviving participants underwent a clinical examination in 2017–2018. Food and nutrient intakes were retrieved from 3-day food diaries in 126 men, and sleep quality and quantity were determined with a questionnaire. Nutritional status was assessed using Mini Nutritional Assessment Short Form (MNA-SF), General Health and Vitality were measured with RAND-36/SF-36 health-related quality of life instrument, and albumin and creatinine levels were analyzed from fasting serum samples.

**Results:**

Mean age of the survivors was 87 years (range 83–99). Self-reported sleep quality and quantity were highly correlated (*p* < 0.001, *η*^2^ = 0.693). Nutritional status (MNA-SF) (*p* = 0.006, *η*^2^ = 0.076), vegetable intake (*p* = 0.030. *η*^2^ = 0.041) and vitality (*p* = 0.008, *η*^2^ = 0.101) were associated with better sleep quality and fish (*p* = 0.028, *η*^2^ = 0.051) intake was associated with longer sleep duration. This association remained after adjusting for age, sleep quality, carbohydrate energy %, and albumin levels.

**Conclusion:**

Healthy nutrition may be an important contributor to sleep hygiene in oldest-old men.

## Key summary points

**Aim:**

To investigate associations of sleep quality and quantity and nutrition in oldest-old men.

**Findings:**

Sleep quality was associated with nutritional status and vegetable intakes, whereas sleep quantity was linked to fish intake.

**Message:**

Healthy nutrition may be an important contributor of sleep hygiene in oldest-old men.

## Introduction

Sleep is a biologic process that is essential for brain function and human physiology including metabolism, appetite regulation, immunity as well as hormonal and cardiovascular systems [1]. Normal sleep may be defined as having sufficient quantity, good quality, and lacking sleep disturbances and disorders [2]. Sleep quality and quantity often decline as people age [3], but how much this is due to intrinsic or extrinsic factors, such as lifestyle, is unclear in very old age.

Nutrition and sleep have been studied in cross-sectional, longitudinal and experimental studies [4–8]. Healthy dietary patterns characterized by abundant intake of fruits, vegetables and legumes have been associated with better sleep quality and less sleep disturbances [5, 6]. Intakes of specific foods, such as fatty fish [7], kiwi fruit [4], and tart-cherry juice [9], and of nutrients (e.g., complex carbohydrates and vitamin D) [8] have also been associated with better sleep in adults. In older people greater dietary diversity [10, 11] and appetite [10] have been linked to better sleep. However, although oldest-old people are a risk group for poor sleep [3], studies on nutrition and sleep among them are very scarce [12]. Therefore, we sought to identify nutrition-related factors that are associated with sleep quality and quantity in oldest-old community-dwelling men.

## Participants and methods

In the Helsinki Businessmen Study (HBS), a socioeconomically homogenous cohort of men–born between 1919 and 1934—has been followed up since the 1960s [13]. From 600 survivors in 2017–2018, a random sub-cohort of 180 home-living men were invited to participate in the clinic visit and 130 men attended (mean age 87 years, range 83–99 years). The main reasons for refusals were poor health, cognitive disorders disease or institutionalization. Examinations included body mass index [BMI, calculated as weight (kg)/height (m) squared], nutritional status using Mini Nutritional Assessment Short form (MNA-SF) [14], and 12-h fasting serum samples analyzed for a variety of parameters at the routine laboratory of the Helsinki University hospital. Although MNA has not been formally validated in Finland, it is widely used as a translation (Nestle…) in Finnish health care. Subjective health and vitality were assessed using scores in appropriate scales (General Health, Vitality) of the RAND-36/SF-36 Health-Related Quality of Life (HRQoL) instrument [15, 16]. Food intakes were retrieved from 3-day food diaries, which a nutritionist checked, verified and asked for clarifications if necessary. The participants were asked about their sleep quality with a question “In your opinion would you classify your sleep quality to be good, average, or poor?”. They were also asked how many hours per night and day they slept. Total diurnal sleeping time was categorized into three groups; < 7, 7–9 and > 9 h of sleep.

We evaluated trend and statistical significance of linearity for both sleep quality and total sleep time using ANOVA for continuous variables, and Cochran Armitage test of categorical variables. Strength of association was calculated using eta square. In addition, we used analysis of covariance (ANCOVA) to investigate independent associations with sleeping time. Covariates were selected based on ANOVA trend analysis and prior research [7, 17, 18] and included age, sleep quality, serum albumin, serum creatinine, fish intake, and carbohydrate energy % of total energy. Statistical analyses were performed using the SPSS statistical program, version 24 (SPSS IBM, Armonk, NY, USA).

### Ethics

All participants signed an informed consent and the study protocol was approved by the Ethics Committee of the Helsinki University Hospital, Department of Medicine. The study is registered with ClinicalTrials.gov identifier: NCT02526082.

## Results

Of the 130 participants who attended the clinic, 126 returned food diaries. Of them, 27% (*n* = 36), 58% (*n* = 75), and 15% (*n* = 22) reported having good, average or poor sleep quality, respectively (Table 1). Self-reported sleep quality correlated with total sleeping time (*p* < 0.001, *η*^2^ = 0.693). Sleep quality showed a linear trend with MNA-SF-assessed nutritional status (*p* = 0.006, *η*^2^ = 0.076) and Vitality score of the RAND-36 (*p* = 0.008, *η*^2^ = 0.101), whereas no significant association was observed with BMI, General Health score of the RAND-36, serum albumin or creatinine levels. Of food intakes, vegetable intake showed a linear trend with higher sleep quality (*p* = 0.030, *η*^2^ = 0.041). Higher intake of sugars showed a non-significant trend for poor sleep quality (*p* = 0.050, *η*^2^ = 0.052). A similar, non-significant trend for poor sleep was observed with higher saturated fatty acid (SFA) (*p* = 0.058, *η*^2^ = 0.035) intake. Other food intakes were not related to sleep quality.

**Table 1:** Characteristics and food intakes according to self-reported sleep quality of the oldest-old men

Characteristics	Self-reported sleep quality				
	Good (*n* = 36)	Average (*n* = 76)	Poor (*n* = 19)	*p* value^1^	*η*^2^
Age, years	87.0 (2.6)	87.5 (3.0)	87.4 (3.1)	0.488	0.006
Total sleep time, h	8.6 (1.3)	8.2 (1.6)	6.8 (1.5)	< 0.001	0.693
MNA-SF, points	13.3 (0.9)	13.1 (1.0)	12.2 (2.2)	0.006	0.076
BMI kg/m^2^	25.9 (2.4)	26.2 (2.7)	24.4 (3.0)	0.151	0.051
Albumin, mmol/L	37.5 (2.9)	38.0 (3.4)	37.5 (1.6)	0.872	0.007
Creatinine, mmol/L	95 (25)	111 (63)	102 (36)	0.397	0.019
General Health, RAND-36, 0–100 points	58 (16)	60 (16)	49 (18)	0.151	0.052
Vitality, RAND-36, 0–100 points	68 (21)	68 (17)	50 (23)	0.008	0.101
Food intakes, g/day					
Vegetables	184.5 (150.3)	157.2 (132.8)	97.0 (58.7)	0.030	0.041
Fruits and berries	147.8 (195.6)	148.0 (144.1)	115.2 (160.2)	0.565	0.005
Nuts	2.9 (6.5)	6.4 (18.6)	3.1 (9.1)	0.736	0.012
Legumes	5.4 (18.0)	7.6 (21.1)	5.6 (12.0)	0.861	0.003
Fish	61.9 (63.2)	63.7 (57.8)	60.3 (52.7)	0.974	0.000
Meat	97.5 (47.1)	106.8 (63.2)	103.4 (47.8)	0.608	0.005
Egg	16.2 (28.9)	16.8 (26.5)	15.7 (35.4)	0.986	0.000
Milk products	283.4 (166.4)	332.1 (246.9)	335.0 (298.3)	0.377	0.009
Cereal products	332.8 (162.9)	346.5 (149.4)	304.0 (130.6)	0.671	0.010
Coffee	230.7 (155.8)	282.2 (207.1)	243.1 (184.4)	0.587	0.015
Tea	118.3 (147.0)	97.1 (155.6)	149.6 (187.0)	0.698	0.014
Alcohol	4.8 (8.4)	4.5 (7.9)	5.0 (7.8)	0.986	0.000
Energy, nutrients per day					
Energy, kcal	1512 (278)	1610 (369)	1641 (439)	0.166	0.017
Protein, g g/kg BW/day Protein E%	72 (17) 0.93 (0.24) 19	74 (23) 0.95 (0.29) 18	73 (25) 1.00 (0.36) 18	0.792 0.439 0.167	0.003 0.006 0.012
Carbohydrates, g Starch Sugar Fiber Carbohydrate E%	164 (39) 82 (27) 23 (12) 22 (7) 44	168 (40) 87 (25) 23 (11) 22 (9) 42	180 (61) 86 (26) 31 (16) 21 (9) 44	0.241 0.486 0.050 0.515 0.854	0.013 0.008 0.052 0.004 0.012
Fat, g SFA MUFA PUFA Fat E%	59 (17) 20 (7) 22 (7) 12 (5) 35	67 (22) 23 (8) 26 (12) 13 (5) 37	66 (22) 23 (8) 25 (12) 11 (5) 36	0.139 0.058 0.174 0.786 0.279	0.030 0.035 0.023 0.012 0.026

Data of continuous variables are mean (SD)

*SD* standard deviation, *MNA-SF* Mini Nutritional Assessment Short Form, *SPPB* Short Physical Performance Battery, *BMI* Body Mass Index, *Alm/m*^*2*^ appendicular lean mass/meter squared, *SFA* saturated fatty acids, *MUFA* monounsaturated fatty acids, *PUFA* polyunsaturated fatty acids

^1^The statistical significance of the hypotheses of linearity was evaluated for a trend using ANOVA for continuous variables and Cochrane Armitage test for categorical variables, *p* value < 0.05 was taken as statistically significant; *η*^2^ = strength of association

For sleep duration, 32%, (*n* = 42), 51% (*n* = 67), and 17% (*n* = 22) of the participants reported sleeping ≤ 7 h, > 7 ≤ 9 h, and > 9 h during night time, respectively (Table 2). Sleeping longer at night was associated with better sleep quality (*p* < 0.001) and higher fish intake (*p* = 0.028, *η*^2^ = 0.051). Other food intakes were not associated with sleep duration. BMI, General Health and Vitality scores of RAND-36, serum albumin and creatinine levels, and other food intakes were not associated with sleep quantity either.

**Table 2:** Characteristics and nutrition intakes according to sleep time at night in oldest-old men

Characteristics	Self-reported sleeping time				
	≤ 7 h (*n* = 42)	> 7–9 h (*n* = 67)	> 9 h (*n* = 22)	*p* value^1^	*η*^2^
Age, years	87.3 (3.3)	87.4 (2.5)	87.0 (3.1)	0.807	0.002
Sleep quality, %					
Good Average Poor	11.9 54.8 33.3	32.8 61.2 6.0	40.9 54.5 4.5	< 0.001	
MNA-SF, points	13.0 (1.6)	13.1 (1.1)	13.1 (0.9)	0.742	0.001
BMI kg/m^2^	25.2 (2.8)	26.2 (2.7)	25.9 (2.4)	0.220	0.027
Albumin, mmol/L	38.2 (2.6)	38.0 (3.1)	36.5 (3.4)	0.059	0.036
Creatinine, mmol/L	102 (27)	102 (30)	121 (108)	0.222	0.020
General health RAND-36, 0–100 points	58 (18)	58 (16)	58 (17)	0.870	0.000
Vitality, RAND-36. 0–100 points	66 (18)	65 (21)	67 (20)	0.906	0.001
Food intakes, g/day					
Vegetables	147 (139)	165 (140)	142 (89)	0.964	0.006
Fruits and berries	131 (147)	133 (149)	196 (211)	0.194	0.022
Nuts	6 (22)	5 (12)	4 (7)	0.536	0.003
Legumes	5 (14)	8 (21)	8 (23)	0.498	0.005
Fish	54 (58)	59 (54)	91 (66)	0.028	0.051
Meat	113 (57)	102 (57)	91 (56)	0.143	0.017
Egg	17 (29)	18 (30)	11 (21)	0.462	0.009
Milk products	136 (212)	335 (208)	331 (342)	0.827	0.000
Cereal products	289 (178)	345 (143)	342 (153)	0.861	0.000
Coffee	251 (165)	247 (163)	334 (291)	0.181	0.028
Tea	120 (180)	113 (158)	84 (113)	0.447	0.006
Alcohol	3 (6)	5 (9)	6 (8)	0.160	0.020
Energy, nutrients per day					
Energy, kcal	1572 (371)	1600 (367)	1577 (319)	0.889	0.001
Protein, g g/kg BW/day Protein E%	69 (28) 0.99 (0.32) 19	74 (19) 0.93 (0.27) 18	75 (25) 0.96 (0.28) 19	0.904 0.628 0.828	0.002 0.008 0.007
Carbohydrates, g Starch Sugar Fiber Carbohydrate E%	165 (45) 85 (23) 24 (14) 22 (9) 42	169 (44) 85 (28) 25 (12) 22 (8) 42	176 (41) 89 (23) 22 (12) 22 (9) 45	0.373 0.530 0.778 0.922 0.262	0.007 0.005 0.006 0.002 0.018
Fat, g SFA MUFA PUFA Fat E%	66 (22) 22 (7) 26 (13) 12 (6) 37	66 (22) 23 (8) 25 (10) 13 (5) 37	59 (15) 20 (8) 22 (8) 11 (3) 33	0.303 0.664 0.223 0.332 0.079	0.017 0.013 0.014 0.016 0.035

Data of continuous variables are mean (SD)

*SD* Standard deviation, *MNA-SF* Mini Nutritional Assessment Short Form, *BMI* body mass index, *kcal* kilocalorie, *BW* body weight, *kg* kilo grams, *SFA* saturated fatty acids, *MUFA* monounsaturated fatty acids, *PUFA* polyunsaturated fatty acids

^1^The statistical significance of the hypotheses of linearity was evaluated for a trend using ANOVA for continuous variables and Cochran Armitage test for categorical variables, *p* value < 0.05 was taken as statistically significant; *η*^2^ = strength of association

In multivariate analyses, sleep duration was associated with sleep quality and fish intake after adjustment for age, albumin level and carbohydrate energy % intake (Table 3).

**Table 3:** ANCOVA models 1 and 2 of associative factors of sleep duration at night time

	*B*	95% confidence interval		*p* value
		Lower bound	Upper bound	
Model 1				
Intercept	6.504	− 1.247	14.255	0.099
Age	− 0.002	− 0.090	0.086	0.988
Sleep quality; good vs. poor	1.793	0.957	2.628	< 0.001
Sleep quality; average vs. poor	1.392	0.641	2.144	< 0.001
Fish intake, g/day	0.007	0.003	0.012	0.002
Adjusted *R*^2^	**0.171**			
Model 2				
Intercept	7.704	4.165	11.242	< 0.001
Sleep quality; good vs. poor	1.822	1.001	2.642	< 0.001
Sleep quality; average vs. poor	1.498	0.753	2.244	< 0.001
Fish intake, g/day	0.007	0.003	0.012	0.001
Albumin, mmol/L	− 0.071	− 0.155	0.013	0.097
Carbohydrate energy % of total energy	0.029	− 0.008	0.066	0.122
Adjusted *R*^2^	**0.197**			

Bold value indicates the amount of observed variation that can be explained by the model’s inputs

## Discussion

In men at age of 83–99 years, sleep quality was associated with nutritional status and vegetable intakes, whereas sleep duration was linked to higher fish consumption. These findings are consistent with the hypothesis that healthy nutrition may contribute to good sleep also in the oldest-old men.

Previous studies have suggested that both sleeping too little or too much a day may be associated with adverse health outcomes [19]. In our study, scores in the General Health and Vitality scales of the RAND-36 instrument were not related to the amount of reported sleep, whereas sleep quality was associated with Vitality scores. Sleep quality and quantity typically decline as people age [3]. Although it is often believed that older people need less sleep than younger ones, a recent study of 40,000 participants suggested that the optimal amount of sleep was similar for all adult age groups. Sleep-related impairments of cognition also seemed to affect all ages equally [20].

Several theories on nutrition and sleep exist. It is, for example, suggested that specific protein-rich foods are particularly good for sleep, because they are sources of amino acid tryptophan—a precursor of sleep-inducing compounds serotonin and melatonin [21]. Furthermore, combining both carbohydrates and protein in the same meal makes tryptophan more available to the brain [22]. In one study, higher complex carbohydrates intake was associated with better sleep. High sugar intake, in turn, had an opposite effect, and was associated with more excessive daytime sleepiness [8]. In our study, the association between sugar intake and poor sleep quality was of borderline significance only (*p* = 0.050), possibly due to insufficient statistical power. Interestingly, higher saturated fatty acid intake also tended to be associated with poor sleep quality (*p* = 0.058). Moreover, higher nutritional status and vegetable intake were associated with better sleep quality. These findings are in agreement with some earlier research where dietary patterns characterized with intake abundant of fruits and vegetables and legumes have been associated with better sleep quality and less sleep disturbances [5, 6] In addition, our finding on fish intake and sleep is in agreement with a study reporting an association between higher fatty fish intake and better sleep quality in participants > 40 years of age [7]. In the Hellenic Longitudinal Investigation of Aging and Diet Study, Mediterranean dietary pattern characterized by high fruit and vegetable, legume and fish intakes was also associated with better sleep quality in people > 75 years of age [23].

The strengths of our study include robust main findings, despite the relatively small sample size, and the fact that to the best of our knowledge, this is the first study to explore the relationship between nutrition and sleep in oldest-old community-dwelling men. A limitation of our study is a crude measurement of sleep characteristics and that data from extensive sleep quality questionnaires or polysomnography were not available. Assessing food intake is challenging. Food diaries are one of the best ways to record food intake in older people. However, they may be affected by considerable under- or overreporting of the foods consumed, which may cause bias. However, every effort was made to ensure the correctness of the dietary records. The participants received written and oral advice beforehand on how to fill in the food records, and phone calls were made afterwards in order to confirm that the dietary information was reported as accurately as possible. Furthermore, the surviving participants of the Helsinki Businessmen Study differ in many ways from the general population thus limiting generalizability. The cross-sectional design of the study is also a limitation and prevents drawing conclusions about temporal relationships between nutritional factors and sleep.

In conclusion, sleep quality and quantity were associated with characteristics of a healthy dietary pattern in oldest-old men living in the community. These findings may extend current nutrition recommendations by emphasizing the importance of abundant intake of fruits and vegetables and regular fish intake for sleep [24].
